# Rejuvenation of leukocyte trafficking in aged mice through PEPITEM intervention

**DOI:** 10.1038/s41514-024-00160-6

**Published:** 2024-07-18

**Authors:** Sophie J. Hopkin, Poppy Nathan, Laleh Pezhman, Jenefa Begum, Julia E. Manning, Lauren M. Quinn, G. Ed Rainger, Helen M. McGettrick, Asif J. Iqbal, Myriam Chimen

**Affiliations:** 1 Institute of Cardiovascular Sciences, University of Birmingham, Birmingham, B15 2TT UK; 2 Institute of Inflammation and Ageing, University of Birmingham, Birmingham, B15 2TT UK

**Keywords:** Cell biology, Biomarkers

## Abstract

Inflammageing leads to uncontrolled leukocyte trafficking in response to inflammatory insults. Here, we used a zymosan-induced peritonitis mouse model on inflammation to investigate the role of the PEPITEM pathway on leukocyte migration in ageing. We then analysed whether PEPITEM could modulate leukocyte migration in older adults. We observed a loss of functionality in the PEPITEM pathway, which normally controls leukocyte trafficking in response to inflammation, in older adults and aged mice and show that this can be rescued by supplementation with PEPITEM. Thus, leading to the exciting possibility that PEPITEM supplementation may represent a potential pre-habilitation geroprotective agent to rejuvenate immune functions.

Growing evidence suggests that the ageing process significantly impacts leukocyte trafficking dynamics during inflammation, thereby compromising protective immunity^1^. We have previously reported that ageing increases homeostatic leukocyte trafficking to the peritoneal cavity in mice through pro-inflammatory mediators and enhanced vascular permeability^1^. Whilst ageing increases neutrophil and monocyte trafficking in response to peritonitis^2,3^, patterns of lymphocyte trafficking are still unknown in this model. Here, we investigated how ageing changes leukocyte trafficking dynamics and the impact of a novel immunopeptide (PEPITEM) has on this using an inflammation model of zymosan-induced peritonitis in young (3-month) and aged (21-month) male mice (Fig. 1A). Zymosan-induced peritonitis typically represents a simplified model of the disease, focusing primarily on the early inflammatory events. It may not adequately capture the later stages of human peritonitis development, including tissue damage and organ dysfunction. Nonetheless, it remains a highly reproducible and robust model, characterised by significant recruitment of various immune cells. We previously identified PEPITEM as a kex regulator of leukocyte trafficking. Indeed, through action of adiponectin on its receptors (AdipoR1/2) on B-cells, PEPITEM is released to then stimulate endothelial production of spingosine-1-phosphate (S1P)^4^. S1P will in turn inhibit leukocyte trafficking through modulation on integrin signalling^4^. We have characterised the decline of the PEPITEM pathway in immune-mediated inflammatory diseases (e.g., RA and T1D)^4^, but lack evidence of its role in aging, particularly how it could influence the inflammatory response in the context of an already inflamed environment as seen with ageing that is associated with low-grade systemic inflammation (inflammageing). In this mouse model, CD45^+^ leukocytes are recruited to the peritoneal cavity at 48 h in both young and old mice as expected (Fig. 1B). Ageing tend to amplify the inflammatory response, with more CD45^+^ leukocytes seen in the aged, inflamed peritoneum compared to young mice (Fig. 1B), in line with earlier studies^2,3^. PEPITEM treatment reduced CD45^+^ leukocyte recruitment to the peritoneal cavity of both young and old mice (Fig. 1B). These findings agree with earlier studies showing efficacy of synthetic PEPITEM in murine models of immune-mediated inflammatory diseases (IMIDs)^4–6^. Further phenotypic analysis revealed a significant reduction in both CD4 and CD8 T-cell subsets with PEPITEM at 48 h in both young and aged mice (Fig. 1C-E). Terminally differentiated CD3^+^KLRG1^+^ T-cells abundance is reportedly increased in the tissues of aged mice^7,8^. Indeed, recruitment of CD3^+^KLRG1^+^ T-cells was low in young mice (Fig. 1F), whereas ageing significantly increased CD3^+^KLRG1^+^ T-cells numbers in the inflamed peritoneum, which was inhibited by treatment with PEPITEM (Fig. 1F). Although circulating numbers of CD3^+^KLRG1^+^ T-cells were comparable between young and aged mice, older mice had significantly more CD3^+^KLRG1^+^ T-cells in the spleen which were mobilised in response to zymosan challenge (Supplementary Figure 2). Moreover, PEPITEM treatment reduced the number of CD4^+^ and CD8^+^ naive (CD62L^+^CD44^-^) and central memory (CD62L^+^CD44^+^) T-cells in young and old mice (Fig. 2A-D). By contrast, PEPITEM only reduced infiltration of CD4^+^ and CD8^+^ effector memory (CD62L^-^CD44^+^) T-cells in the young mice, but not the aged (Fig. 2E-F). In the B-cell compartment, CD19^+^ B-cells numbers were higher in the inflamed peritoneum of young mice^9^, which was inhibited by PEPITEM (Fig. 2G). By contrast, the number of CD19^+^ B-cells remained unchanged by treatment (zymosan or PEPITEM) in the aged mice (Fig. 2G). We observed a significant increase in age-associated B-cells (CD19^+^CD21^-^CD93^-^CD23^-^CD43^-^) in response to zymosan in both young and aged mice, with PEPITEM significantly reducing this (Fig. 2H). Taken together, these data suggest that PEPITEM can control the magnitude of an inflammatory response even in the ageing micro-environment, where low-grade chronic inflammatory phenotypes normally prevail and hinder efficient resolution^1^.

**Fig. 1:**
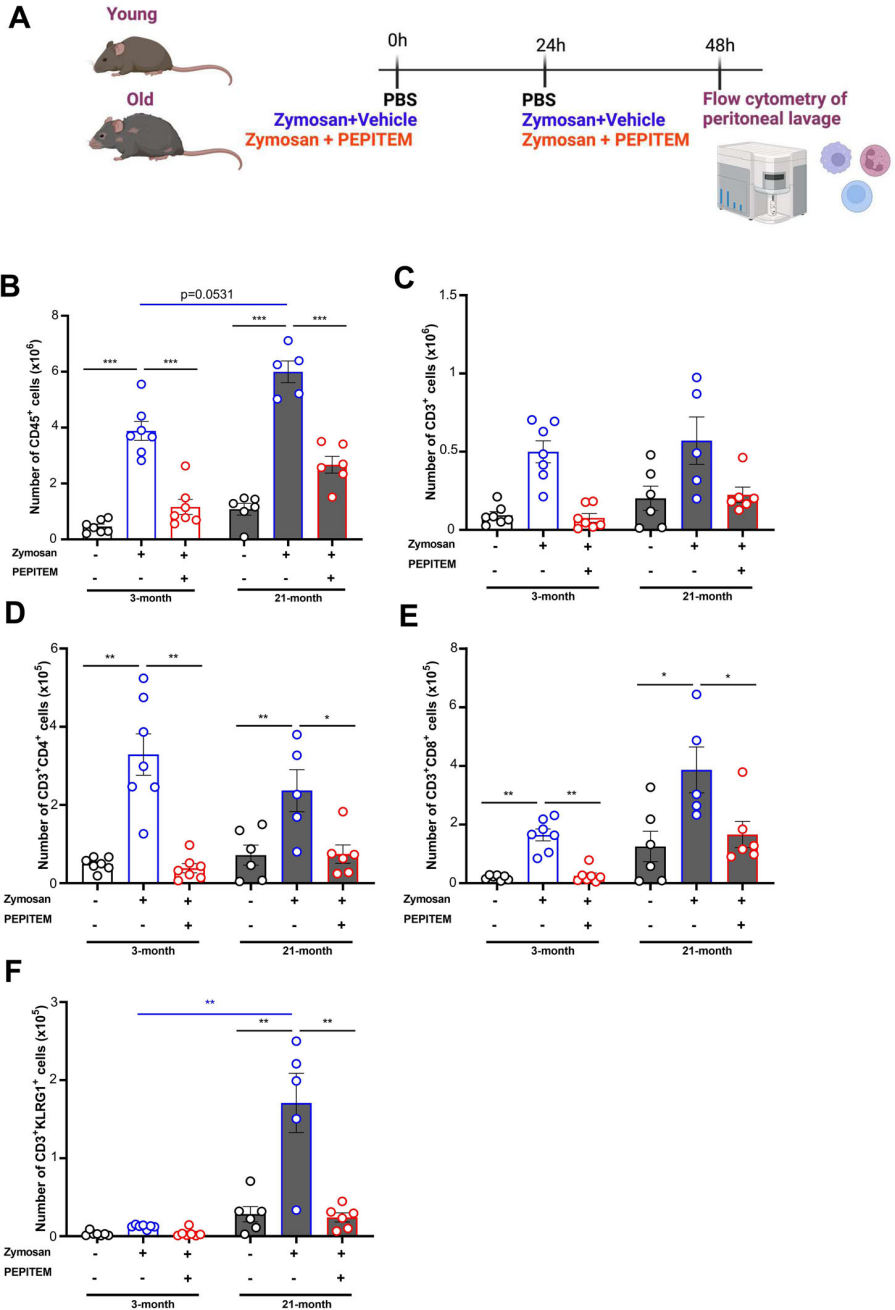
PEPITEM regulates T-cell trafficking in young and old mice. **A** Schematic representation of zymosan-induced peritonitis (blue) for young (3-month; white) and aged (21-month; grey) mice treated without (black) or with PEPITEM (red) (created by biorender.com). The total number of peritoneal **B** CD45^+^ leukocytes, **C** CD3^+^ T-cells, **D** CD3^+^CD4^+^ T-cells, **E** CD3^+^CD8^+^ T-cells and **F** CD3^+^KLRG1^+^ T-cells were quantified using flow cytometry. Data are mean ± SEM for *n* = 2 independent experiments, using *n* = 7 and *n* = 5–6 mice per group for young and old mice. *p < 0.05 and **p < 0.01 by Bonferroni post-test.

**Fig. 2:**
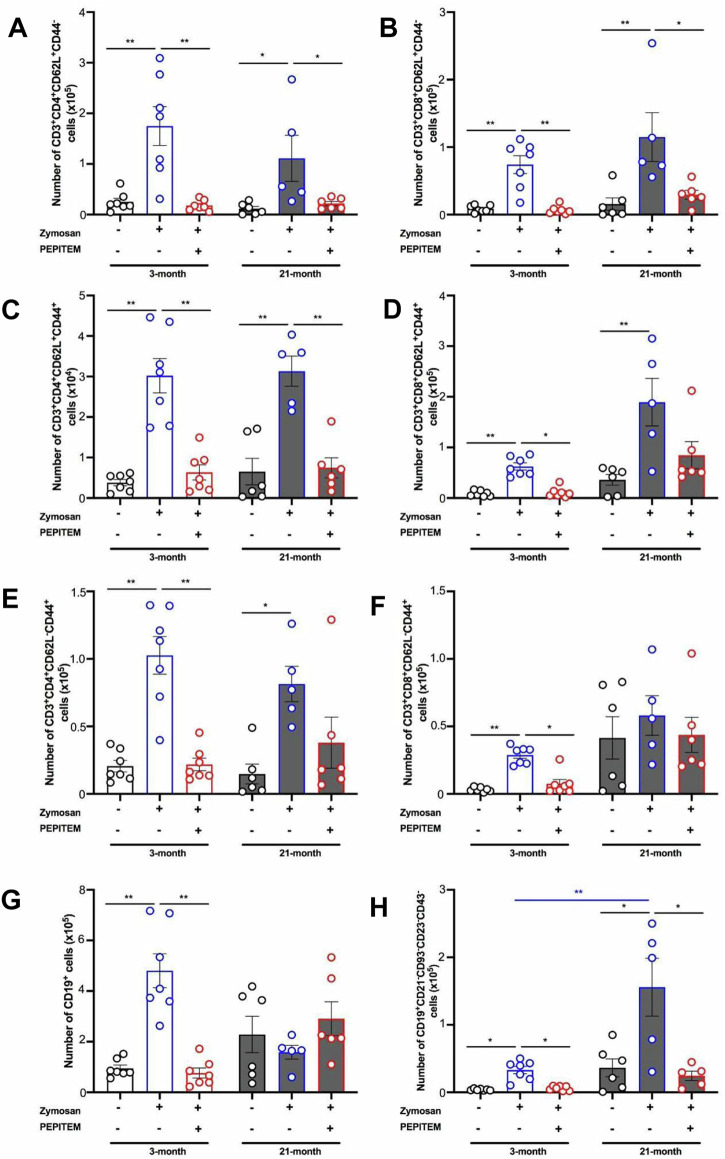
PEPITEM regulates infiltration of T- and B-cell subsets in young and old mice. T- and B-cell subpopulations were measured in the peritoneal lavage fluid of zymosan-induced peritonitis (blue) from young (3-month; white) and aged (21-month; grey) mice treated without (black) or with PEPITEM (red). Total number of peritoneal CD3^+^ **A** CD4^+^CD62L^+^CD44^-^ naive, **B** CD8^+^CD62L^+^CD44^-^ naive, **C** CD4^+^CD62L^+^CD44^+^ central memory, **D** CD8^+^CD62L^+^CD44^+^ central memory, **E** CD4^+^CD62L^-^CD44^+^ effector memory and **F** CD8^+^CD62L^-^CD44^+^ effector memory T-cells, **G** CD19^+^ B-cells and **H** CD19^+^CD21^-^CD93^-^CD23^-^CD43^-^ age-associated B-cells were quantified using flow cytometry. Data are mean ± SEM for *n* = 2 independent experiments, using *n* = 7 and *n* = 5-6 mice per group for young and old mice. *p < 0.05 and **p < 0.01 by Bonferroni post-test.

We, and others, have shown that the adiponectin-PEPITEM pathway is dysregulated in age-related IMIDs, such as rheumatoid arthritis, type 1 diabetes and lupus^4–6^. Very few groups have investigated the effect of ageing on lymphocyte adhesion and transendothelial migration in vitro, reporting no difference between young and old cohorts^10–12^. Here, we assessed the functionality of the adiponectin-PEPITEM pathway in older adults in vitro using a static migration assay modelling the recruitment of lymphocytes in response to inflammation^4^. In agreement with the literature, similar levels of adhesion to cytokine-stimulated endothelial cells were seen using lymphocytes from young (under 40 years old) and old adults (over 65 years old) (Fig. 3A). As previously reported^4^, both adiponectin and PEPITEM treatments reduced the transendothelial migration of lymphocytes from young donors (Fig. 3B). Interestingly, aged lymphocytes were unable to respond to adiponectin, but this defect was rescued by exogenous supplementation with PEPITEM (Fig. 3B). Collectively, these data indicate that the PEPITEM pathway is dysfunctional in older adults, but can be rescued via PEPITEM supplementation.

**Fig. 3:**
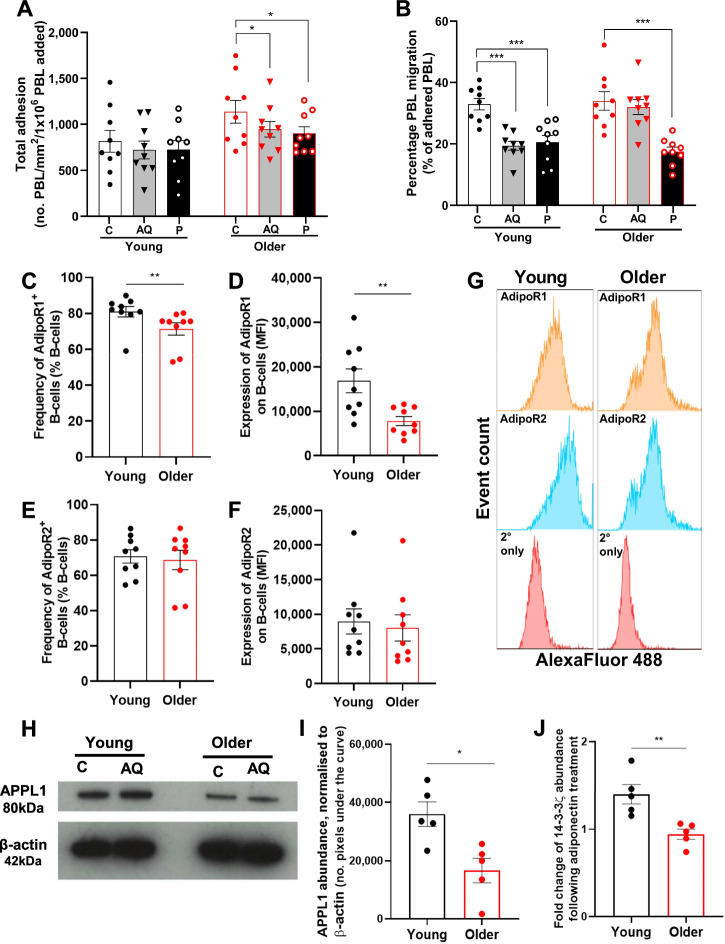
PEPITEM restores endogenous regulation of lymphocyte migration in older adults. PBL from young (black, *n* ≥ 9) and older (red, *n* ≥ 9) donors were left untreated (control, C) or treated with adiponectin (AQ) or PEPITEM (P) prior to addition to cytokine-stimulated endothelium. PBL **A** adhesion or **B** transmigration expressed as the number of cells/mm2/number of cells added or the percentage of adhered cells that had transmigrated, respectively. Adiponectin receptor 1 **C–D** and **E–F** 2 analysed as **C, E** frequency of B-cells positive for expression or **D–F** median fluorescence intensity (MFI; *n* = 9). **G** Representative histograms of AdipoR1 and 2 expression on young and older B-cells. **H–J** B-cells from young (black) and older (red) donors were left **C-G, I** untreated or **H, J** treated with adiponectin for 15 minutes. **H** APPL-1 protein expression was assessed by western blot in B-cell lysates and **I** normalised to actin loading control (*n* = 5). **J** Fold-change in 14-3-3ζ abundance in adiponectin-treated B-cells normalised to β-actin (*n* = 5). Data are mean ± SEM. *p < 0.05, **p ≤ 0.01 and ***p ≤ 0.001 by (**A, B**) Dunnett’s post-test or (**C–D**, **G–H**) unpaired t-test.

Adiponectin predominantly binds and signals through adiponectin receptor 1 and 2 (AdipoR1/2)^13,14^, although it can also interact with vascular T-cadherin^15,16^. Surface expression of AdipoR1/2 on B-cells positively correlates with circulating PEPITEM concentrations^4^. In our aged cohort, AdipoR1^+^ B-cells frequency was lower and surface expression was reduced (~50%) (Fig. 3C-D, G). AdipoR2 expression did not change with age (Fig. 3E-G). By contrast, reduced AdipoR1/2 has been reported on B-cells from patients with rheumatoid arthritis and type-1-diabetes and was linked to a loss of responsiveness to adiponectin^4^. Given that B-cells still express AdipoR1/2, this is unlikely to account for the loss of adiponectin response in ageing. We subsequently investigated whether known signalling pathways downstream of AdipoR1/2 were also changed with age and could contribute to the loss of control of PEPITEM on lymphocyte recruitment.

Age alters the expression, activation and/or phosphorylation rates of several signalling components downstream of AdipoR1/2^17–20^. However, none of these studies looked at the adiponectin signalling cascade in immune cells. APPL1 is the proximal adaptor protein that interacts with both adiponectin receptors and is required for their signalling^21,22^. APPL1 facilitates the recruitment and activation of downstream signalling molecules, such as AMP-activated protein kinase (AMPK) and p38 mitogen-activated protein kinase (p38 MAPK), which are essential in adiponectin-mediated anti-inflammatory responses^23–25^. Therefore, to determine the molecular mechanism by which the adiponectin-PEPITEM pathway is dysregulated in older adults, we measured the expression of APPL1 in B-cells. APPL1 levels in B-cells from older adults were significantly lower compared to young individuals (Fig. 3G, H**;** Supplementary Table 1), which is consistent with single-cell RNA sequencing analysis that demonstrated a notable decrease in *APPL1* gene expression in circulating B-cells among older (>60 years) adults when compared to younger adults (<45 years)^26^. Similarly, expression of the 14-3-3ζ gene (*YWHAZ;* parent protein for PEPITEM) was significantly lower in naive B-cells from older (>60 years) adults compared to younger individuals^27^. Lower APPL-1 in aged B-cells resulted in a significant reduction of 14-3-3ζ produced by aged B-cells in response to 15-minute stimulation with adiponectin respectively when compared to young donors (Fig. 3I). Thus, age-related changes in the downstream signalling through the adiponectin receptors likely drive the loss of response to adiponectin and dysregulation of lymphocyte transendothelial migration.

While lifespan is increasing at globally, healthspan is not keeping pace. There is an imminent necessity for innovative geroprotective agents that enhance healthspan to reduce the burden on the healthcare systems and the wider global economy. Current therapies for IMIDs and age-related conditions target inflammatory/pathological mediators driving disease. In contrast, PEPITEM represents a unique approach as it not only dampens excessive inflammation, but supports the maintenance of immune homeostasis. Previous studies have established PEPITEM as a regulator of T-cell migration to sites of inflammation in various models of IMIDs, including endotoxin-induced uveitis, a virally induced model of Sjӧgren’s syndrome, a MRL/lpr mouse model of systemic lupus erythematosus (SLE), and obesity^4–6^. Our study builds upon these findings by demonstrating that PEPITEM treatment effectively reduces T-cell recruitment to the peritoneal cavity during zymosan-induced peritonitis in young mice. Significantly, our findings extend the therapeutic potential of PEPITEM to aged mice, where it also limits T-cell infiltration. In the context of aged individuals, we demonstrate that the PEPITEM-adiponectin pathway is defective. Supplementation with exogenous PEPITEM could fully restore the regulation of lymphocyte migration in ageing population. Although the current study focused exclusively on male subjects, given the reported differences in inflammatory responses between males and females^9,28^, it is crucial to investigate the effects of PEPITEM in both sexes for a comprehensive understanding. Collectively these findings suggest the potential therapeutic application of PEPITEM as a potential pre-habilitation geroprotective agent that will facilitate and rejuvenate immune responses to diverse stimuli in the aged population.

## Methods

### Ethics Approval Statement

Animal studies were regulated by the Animals (Scientific Procedures) Act 1986 of the United Kingdom and performed under Personal Project Licence P379E5607. Approval was granted by the University of Birmingham’s Animal Welfare and Ethical Review Body and all ethical guidelines were adhered to whilst carrying out this study. All participants were provided informed written consent before donating blood samples. Ethical approval was awarded by the University of Birmingham Local Ethical Review Committee (ERN_12-0079).

### Mice

Experiments were conducted in accordance with UK Home Office regulations and appropriate ethics. Six to eight-week-old and 19-month-old male C57Bl/6 J wild type (WT) mice were purchased from Charles River and were housed at the University of Birmingham animal unit with free access to food and water. The attrition rate for the aged mice was 20%, which was accounted for during the purchasing of the mice. Mice were maintained in an SPF environment, determined by quarterly health screening of the unit using Federation of European Laboratory Animal Science Associations approved methods. Mice were fed the rodent 5LF2 diet (IPS LabDiet) and were housed in the animal facility for at least 2 months prior to experimentation. Environmental conditions were: 21 ± 2 °C, 55 ± 10% relative humidity and a 12 h light-dark cycle.

### Induction of zymosan-induced peritonitis

Peritonitis was induced by intraperitoneal injection (IP) of 0.1 mg zymosan A (Merck, UK) from Saccharomyces cerevisiae, as previously described^29,30^. Mice received an IP injection of the vehicle (PBS; Sigma-Aldrich), or 300 µg pegylated PEPITEM, SVTEQGAELSNEER-[PEG6]-amide (Cambridge Research Biochemicals, CRB), concurrently with zymosan administration, and again at the 24-hour time-point (Chimen et al., 2015). Researchers were blinded to treatment groups and animals were allocated randomly. After 48 hours, mice were sacrificed by terminal anaesthesia (isoflurane) and conformation by cervical dislocation and tissues were collected as described below.

### Flow cytometry on peritoneal lavage fluid

The peritoneal cavity was lavaged with ice-cold 5 mM EDTA. Peritoneal lavage fluid (PLF) was centrifuged at 400 *g* for 5 minutes: supernatant was stored at -80 °C and cells were resuspended in MACS buffer (0.1 mM EDTA, 0.6% BSA in PBS, all from Sigma-Aldrich). Samples were blocked with FcR blocker (Miltenyi Biotec) prior to staining with the following antibodies for 20 minutes at 4 °C, after which samples were washed and fixed with 2% formaldehyde: anti-CD3 PECy7 (clone 145-2c11), anti-CD4 eFluor450 (clone GK1.5), anti-CD8 PE-TexasRed (clone 5H10), anti-CD44 FITC (clone IM7), anti-CD25 AF700 (clone PC61.5), anti-KLRG1 APC-eFluor780 (clone 2F1), anti-CD62L PE (clone MEL-14), anti-CD19-APC (clone 1D3), anti-CD45 APC-CY7 (clone 104), F4/80 FITC (clone BM8), anti-CD11c PE-Cy7 (clone N418), anti-gp38 PE (clone 8.1.1; all from Thermofisher); anti-CD45.2 BV605 (clone 104), anti-CD23 BV421 (clone B3B4), anti-CD93 BV650 (clone AA4.1), anti-CD43 PerCP-Cy5.5 (clone S7), anti-CD21/35 PE (clone 7G6), anti-Siglec F PE-CF594 (clone E50-2440), Ly6G APC (clone 1A8; all from BD) (Working dilutions and catalogue numbers are provided Supplementary Table 2). Compensation controls were generated using cells isolated from the spleen. Immediately prior to analysis CountBright beads (Invitrogen) and Zombie Aqua (Biolegend) were added and samples were acquired using Fortessa-X20. Data were analysed offline using FlowJo (V-10.2.6) using the flow cytometry gating strategy depicted in Supplementary Figure 1.

### Isolation of lymphocyte subpopulations

Ethical approval for this project was awarded by the University of Birmingham. Local Ethical Review Committee (ERN_12-0079). Healthy volunteers recruited for this study were required to be >18 years old, to not have any active infections or immune-related diseases, and not to have had any vaccines <3 month prior to sample collection. Human blood from young (23-37 years) and older (60-78 years) healthy men was collected into EDTA-coated vacuettes. PBMC were isolated from blood using a histopaque (Sigma-Aldrich) gradient, as previously described^31^. The PBMC pellet was resuspended in medium 199 (M199; Gibco) with 0.15% BSA or in MACS buffer. To remove monocytes, PBMC were incubated on plastic (37 °C, 5% CO2) for 45 minutes^32^. The non-adherent PBL were then collected, washed with M199 0.15% BSA and centrifuged at 300 *g* for 5 minutes. Pelleted PBL were re-suspended to 1 × 10^6^ cells/ml prior to the static adhesion assay. Cells were treated with or without 10 µg/ml adiponectin (Enzo Life Sciences, UK) for 1 hour or 20 ng/ml PEPITEM (CRB) immediately prior to use.

To isolate B-cells, the EasySep Human B-cell Enrichment kit (StemCell, UK) was used according to the manufacturer’s instructions. Isolated B-cells (5×10^5^) were resuspended in MACS buffer and treated with or without 10 µg/ml recombinant adiponectin for 15 minutes under agitation at room temperature. Samples were then lysed using the EasyPep Mini MS Sample Prep kit’s lysis buffer (ThermoFisher) containing 0.5kU universal nuclease and 10% protease inhibitor cocktail (Sigma-Aldrich) and subjected to liquid chromatography mass spectrometry (LC/MS) or western blot analysis.

### Static adhesion assay

Human dermal blood endothelial cells (HDBEC; 2×10^5^ cells/well; Lot #423Z004, Promocell) were seeded onto collagen gels as previously described^33^, and cultured overnight at 37 °C, ^31^5% CO_2_. HDBEC were stimulated with 100U/ml TNFα (R&D Systems, UK) and 10 ng/ml IFNγ (PeproTech, UK) for 24 hours prior to washing and addition on PBL. PBL were either untreated, pre-treated with 10 µg/ml recombinant adiponectin (Enzo Life Sciences, UK) for 1 hour prior to the assay, or treated with 20 ng/ml PEPITEM (CRB, UK) during the assay. PBL were permittedto migrate for 20 minutes (37 °C, 5% CO_2_). Non-adherent PBL were then removed by gentle washing with M199 supplemented with 0.15% BSA. Wells were imaged using a phase-contrast Olympus IX70 microscope (Olympus, UK) and digitised images analysed off-line using Image-Pro 6.2 software (Media Cybernetics; Maryland, USA) as previously described^31^. Total PBL adhesion was expressed as cells per mm2 per number of PBL added. PBL transendothelial migration was calculated as a percentage of the total number of adhered cells.

### Flow cytometric analysis of human lymphocytes

All samples were blocked with FcR blocker (Miltenyi Biotec) prior to staining with the following antibodies for 20 minutes at 4 °C: anti-CD19 PECy7 (clone HIB19, ThermoFisher), rabbit anti-AdipoR1 polyclonal and anti-AdipoR2 polyclonal (both from Phenoix Pharmaceuticals), and goat anti-rabbit IgG AF488 secondary (ThermoFisher). Compensation controls were generated using cells. Immediately prior to analysis CountBright beads (Invitrogen) and Zombie Aqua (Biolegend) were added and samples were acquired using Fortessa-X20. Data were analysed offline using FlowJo (V-10.2.6). Background staining in isotype control samples was subtracted from the signal for the protein of interest. Adiponectin receptor expression was expressed as frequency of B-cells positive for staining or as median fluorescence intensity (MFI) of positive cells.

### Western blot

B-cell proteins (5 µg) were diluted 1:5 with Laemmli Sample buffer (BioRad) and incubated at 95 °C for 10 minutes. Samples were then loaded into a NuPAGE 4-12% Bis-Tris mini protein gel (Invitrogen) and electrophoresed at 100 V for 90 minutes. Proteins were transferred onto a PVDF Midi 0.2 mm membrane (BioRad) using the Trans Blot Turbo system (BioRad) for 10 minutes at 25 V. The membrane was then blocked with 5% milk diluted in PBS-Tween 0.1% (blocking buffer; reagents from Sigma-Aldrich) for 1 hour at RT under agitation. The membrane was then incubated with 1:4,000 rabbit anti-APPL1 (12639-1-AP, ProteinTech) overnight at 4 °C under agitation. The membrane received three rounds of 10-minute washes using 0.1% PBS-Tween (PBST), and was then incubated with 1:10,000 goat anti-rabbit IgG peroxidase conjugate (401393, EMD Millipore Corp, UK) for 90 minutes at 4 °C under agitation. After washing, Clarity Western ECL chemi reagent substrate (BioRad) was added to the membrane for 1 minute. The membrane was then exposed to X-ray film for 30 seconds and developed using the Compact X4 Automatic X-ray Film Processor (Xograph, UK). The membrane was then incubated with 20 ml Restore Western Blot Stripping Buffer (Thermo Scientific) at 37° for 30 minutes under agitation to strip antibodies from the membrane. The membrane was then re-probed with 1:4,000 rabbit anti-actin (A2066, Sigma-Aldrich) and 1:10,000 goat anti-rabbit IgG peroxidase conjugate antibodies in a similar manner to above, and exposed to X-ray film for 1 second. Blots were scanned and analysed offline using ImageJ (version 2.1.0, Cambridge). To quantify protein abundance, the area under the peak method was employed^34^. Briefly, a region of interest (ROI) was drawn around each protein band and grey-pixel intensity plots were generated by ImageJ. A horizontal line was drawn underneath the peak to eliminate background noise, and the area under the peak was measured. To normalise the data according to total protein abundance of the sample, the area under the peak data for the actin bands were normalised to the lowest actin value, and the corresponding normalisation ratios were applied to the APPL1 data. The data are presented as APPL1 abundance normalised to actin loading control (Supplementary Figure 3- original blots).

### LC-MS

B-cells were prepared for LC/MS analysis using the EasyPep Mini MS Sample Prep kit (ThermoFisher) according to the manufacturer’s instructions. Protein concentration of the lysates was quantified using the Pierce BCA Protein Assay kit (ThermoFisher) according to the manufacturer’s instructions. B-cell lysate protein (70 μg/sample) was then reduced, alkylated, digested and cleaned using the EasyPep Mini MS Sample Prep kit (ThermoFisher). Following elution, peptides were placed into a Concentrator 5301 vacuum centrifuge (Eppendorf, UK) and dried overnight prior to LC/MS analysis at the Advanced Mass Spectrometry facility (Birmingham, UK). Peptides were resuspended in 0.1% formic acid diluted in water, and injected into the Dionex Ultimate 3000 liquid chromatography system and Thermo Q Exactive HF Orbitrap mass spectrometer (ThermoFisher). A gradient of 3-44% v/v acetonitrile in 0.1% formic acid was run at 350 nl/min for 30 minutes to facilitate the separation of the peptides. A mass spectrometry survey of 380 to 1800 m/z was performed and automatic gain control for selecting ions was set at MS1 3 × 106 and MS2 1 × 105. The m/z peaks generated by Thermo Xcalibur software (ThermoFisher) were then identified using the Proteome Discoverer 2.2 software (ThermoFisher) with the Uniprot protein database. The relative abundance of proteins in adiponectin-treated B-cell samples compared to untreated B-cell control sample for a single donor was calculated and expressed as fold change.

### Statistical analysis

Data were analysed using GraphPad Prism and presented as mean ± SEM for n mice per group for n independent experiments. Normality was assessed using the Shapiro-Wilk test. Univariate analysis was performed using paired or unpaired t-test. Multivariate analysis was performed using ANOVA, with Bonferroni or Dunnett’s post-test. *p* < 0.05 was deemed statistically significant.

### Supplementary information

Supplementary Information

## Data Availability

Data are available upon reasonable request. All data relevant to the study are included in the article or uploaded as supplementary information.
