# Supplementary material for: Rejuvenation of leukocyte trafficking in aged mice through PEPITEM intervention
Source: NPJ Aging. 2024 Jul 18;10(1):33. doi: 10.1038/s41514-024-00160-6 (PMC11258258; doi:10.1038/s41514-024-00160-6)
Supplement: Supplementary file 1 — Supplementary Information [file 41514_2024_160_MOESM1_ESM.pdf]

Rejuvenation of leukocyte trafficking in aged mice through PEPITEM intervention

Sophie J. Hopkin[1], Poppy Nathan[1], Laleh Pezhman[1], Jenefa Begum[1], Julia E. Manning[2], Lauren M. Quinn[1], G. Ed Rainger[1], Helen M. McGettrick[2§#], Asif J. Iqbal[1§#] and Myriam Chimen[2§#]

[1]Institute of Cardiovascular Sciences, University of Birmingham, Birmingham, B15 2TT, UK.
[2]Institute of Inflammation and Ageing, University of Birmingham, Birmingham, B15 2TT, UK.

[§]Authors contributed equally to this research.

Keywords: Inflammation, ageing, T-cells, PEPITEM, sexual dimorphism, leukocyte trafficking

Correspondence:
[#]Dr Myriam Chimen, Institute of Inflammation and Ageing, University of Birmingham, Birmingham, B15 2TT, UK.
Email: m.chimen@bham.ac.uk
[#]Dr Asif. J. Iqbal, Institute of Cardiovascular Sciences, University of Birmingham, Birmingham, B15 2TT, UK.
Email: a.j.iqbal@bham.ac.uk
[#]Dr Helen M. McGettrick, Institute of Inflammation and Ageing, University of Birmingham, Birmingham, B15 2TT, UK.
Email: h.m.mcgettrick@bham.ac.uk

Supplementary Figures:

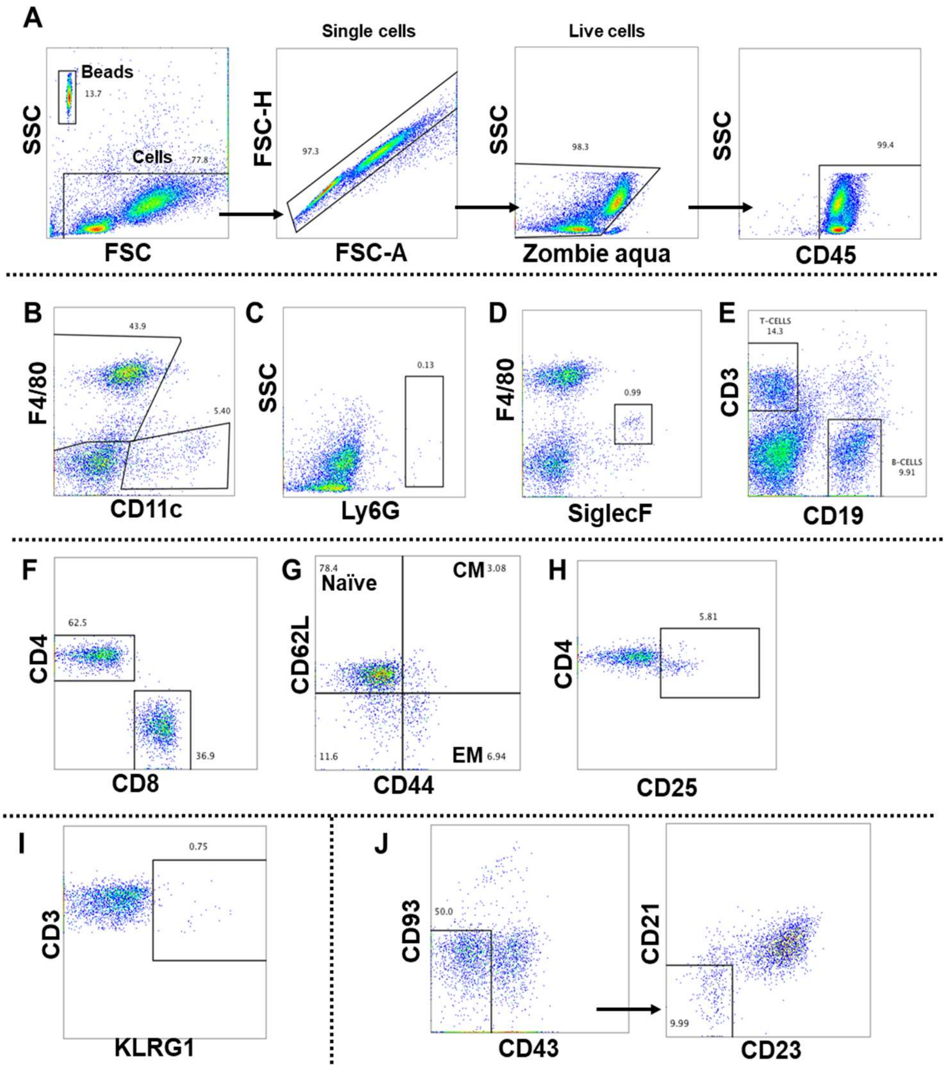

Supplementary Figure 1 – Flow cytometry gating strategy for mouse tissues

Flow cytometry gating strategy used to phenotype and quantify leukocytes within the peritoneal exudate of naïve young (3 month) and aged (21 month) male C57Bl6 mice. Gating strategy to define **(A)** beads and single, live CD45^{+} leukocytes. Gating strategy to identify **(B)** F4/80^{hi} macrophages, CD11c^{+} dendritic cells, **(C)** Ly6G^{+} neutrophils, **(D)** SiglecF^{+} eosinophils, **(E)** CD3^{+} T-cells and CD19^{+} B-cells within the leukocyte population. **(F)** T-cells were distinguished based on CD4 and CD8 expression, and then based on **(G)** CD62L and CD44 expression to identify naïve (CD62L^{+}CD44^{-}), central memory (CM; CD62L^{+}CD44^{+}), and effector memory (EM; CD62L^{-}CD44^{+}) subsets. **(H)** Regulatory T-cells were identified as CD3^{+}CD4^{+}CD25^{+} cells. **(I)** Senescent T-cells were identified as CD3^{+}KLRG1^{+} cells. **(J)** Age-associated B-cells were identified as CD19^{+}CD93^{-}CD43^{-}CD21^{-}CD23^{-} cells.

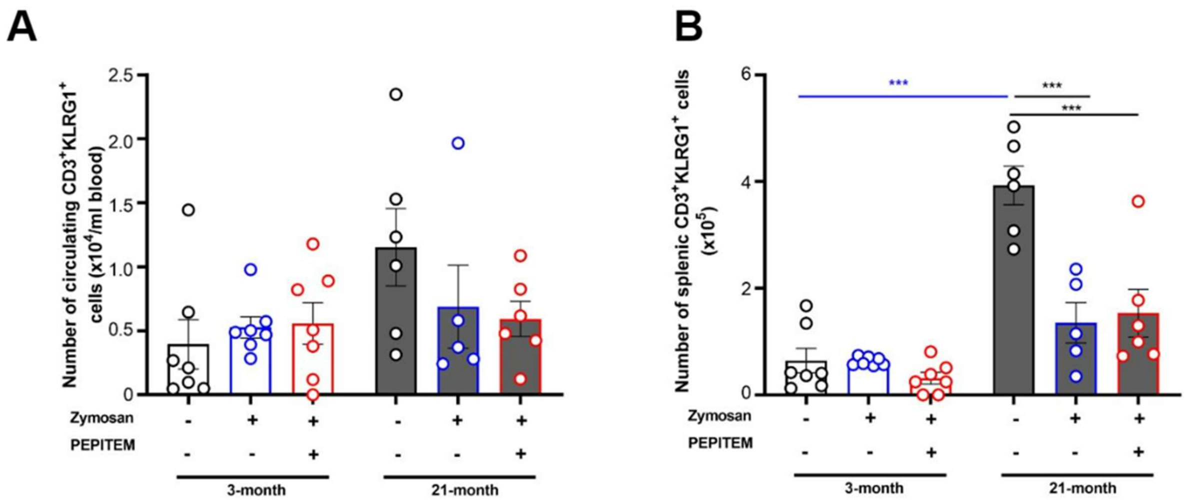

Supplementary Figure 2: Zymosan challenge mobilises CD3$^+$KLRG1$^+$ cells from the spleen in aged mice. CD3$^+$KLRG1$^+$ cells were quantified in the (A) circulation and (B) spleens of vehicle-treated (black) or zymosan-induced peritonitis young (3-month; white) and aged (21-month; grey) mice treated without (blue) or with PEPITEM (red). Total number of circulating and splenic CD3$^+$KLRG1$^+$ cells were quantified using flow cytometry. Data are mean ± SEM for n=2 independent experiments, using n=7 and n=5-6 mice per group for young and old mice. ***$p<0.001$ by Bonferroni post-test.

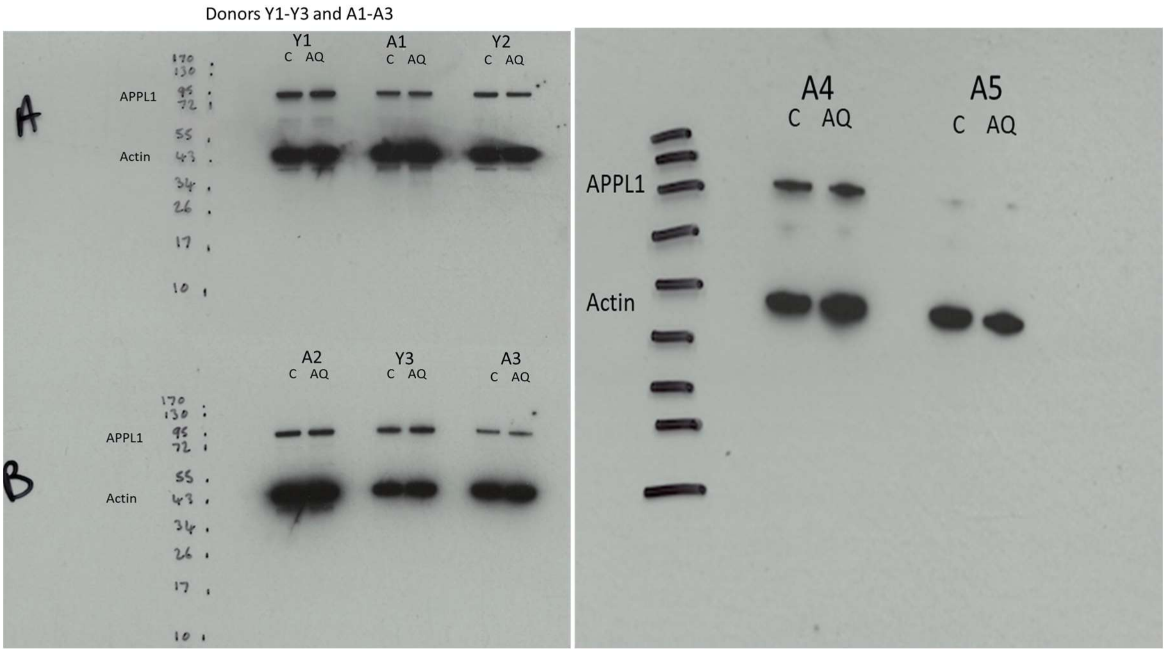

Supplementary Figure 3: Western Blots APPL1. APPL-1 protein expression was assessed by western blot in B-cell lysates and normalised to actin loading control (n=5).

		Area under the peak value (pixels)	
		APPL1	β-actin
Young donors	1	42317.8185	49213.2785
	2	28715.0575	60187.1735
	3	34742.59	50915.517
	4	43308.1595	44684.3185
	5	38718.6285	57932.0775
Older donors	1	24126.7245	53322.5165
	2	34791.963	85691.349
	3	18288.7395	66385.569
	4	20802.9005	39700.2515
	5	1234.83	35631.71

Supplementary Table 1: Quantification of APPL1 and β-actin protein bands obtained from western blotting B-cell lysates. Lysates of untreated B-cells derived from young and older donors were subjected to western blot analysis. Quantification of the APPL1 and β-actin protein bands was performed using ImageJ, where regions of interest were drawn around the bands and the 'area under the peak' values were extracted. The raw 'area under the peak values' are presented in this table.

Antibodies							
**Target**	**Clone**	**Conjugate**	**Isoform**	**Manufacturer**	**Catalogue #**	**Concentration (µg/ml)**	**Dilution**
CD45.2	104	BV605	IgG2a, κ	BioLegend	109841	200	1:100
CD45.2	104	APC-eFluor 780	IgG2a, κ	ThermoFisher	47-0454-82	200	1:200
CD3ξ	145-2C11	PECy7	IgG	ThermoFisher	25-0031-82	200	1:50
CD4	GK1.5	eFluor450	IgG2b, κ	ThermoFisher	48-0041-82	200	1:100
CD8	5H10	PE-Texas Red	IgG2b	ThermoFisher	MCD0817	N/A	1:200
CD62L	MEL-14	PE	IgG2a, κ	ThermoFisher	12-0621-82	200	1:500
CD44	IM7	FITC	IgG2b, κ	ThermoFisher	11-0441-82	500	1:500
CD25	PC61.5	Alexa fluor 700	IgG1, κ	ThermoFisher	56-0251-82	200	1:50
KLRG1	2F1	APC-eFluor 780	IgG2a, κ	ThermoFisher	47-5893-82	200	1:100
CD19	1D3	APC	IgG2a, κ	ThermoFisher	17-0193-82	200	1:50
CD21/35	7G6	PE	IgG2b, κ	BD Biosciences	552957	200	1:100
CD23	B3B4	BV421	IgG2a, κ	BD Biosciences	562929	200	1:100
CD93	AA4.1	BV650	IgG2b, κ	BD Biosciences	563807	200	1:100
CD43	S7	PerCPCy5.5	IgG2a, κ	BD Biosciences	562865	200	1:100
F4/80	BM8	FITC	IgG2b	ThermoFisher	MA5-16628	100	1:50
CD11c	N418	PECy7	IgG	ThermoFisher	25-0114-82	200	1:50
Siglec F	E50-2440	PE-CF594	IgG2a, κ	BD Biosciences	562757	200	1:50
Ly6G	1A8	APC	IgG2a, κ	BD Biosciences	560599	200	1:50
CD3ξ	145-2C11	PE	IgG	ThermoFisher	12-0031-82	200	1:100
CD3ξ	145-2C11	FITC	IgG	ThermoFisher	11-0031-82	500	1:100
CD45.2	30-F11	Alexa fluor 700	IgG2b, κ	ThermoFisher	56-0451-82	200	1:100
CD3ξ	17A2	BV650	IgG2b, κ	BD Biosciences	100229	N/A	1:100
CD3ξ	17A2	BV605	IgG2b, κ	BD Biosciences	100237	N/A	1:100
CD3ξ	145-2C11	PerCPCy5.5	IgG	ThermoFisher	45-0031-82	200	1:100

Primary antibody	Isotype	Manufacturer	Catalogue #	Concentration (µg/ml)	Dilution
CD25 Alexa fluor 700	IgG1, κ Alexa fluor 700	ThermoFisher	56-4301-80	200	1:50
KLRG1 APC-eFluor 780	IgG2a, κ APC-eFluor 780	ThermoFisher	47-4321-82	200	1:100
CD21/35 PE	IgG2b, κ PE	ThermoFisher	12-4031-82	200	1:100
CD23 BV421	IgG2a, κ BV421	BD Biosciences	562602	200	1:100
CD93 BV650	IgG2b, κ BV650	BD Biosciences	563233	200	1:100
CD43 PerCPCy5.5	IgG2a, κ PerCPCy5.5	ThermoFisher	45-4321-80	200	1:100

Supplementary Table 2: List of primary mouse antibodies and corresponding isotype controls.
